# How useful are randomized controlled trials in a rapidly changing world?

**DOI:** 10.1017/gmh.2015.29

**Published:** 2016-03-02

**Authors:** A. Perez-Gomez, J. Mejia-Trujillo, A. Mejia

**Affiliations:** 1 Corporación Nuevos Rumbos, Colombia; 2 Instituto de Investigaciones Cientificas y Servicios de Alta Tecnologia (INDICASAT), Panama

The term *evidence-based* to label mental health interventions is increasingly common. Fast developments in mental health and social sciences have prompted policy makers to fund and implement only those interventions that have been evaluated through rigorous experimental studies such as randomized controlled trials (RCTs; e.g. Institute of Medicine, 2009; Centers for Disease Control and Prevention, 2011). While in the last 50 years significant investments have been made in high-income countries to establish the evidence of many intervention packages, very little has been done in low- and middle-income countries (LMICs). As a response, there is now a global mental health movement for increasing evaluations and access to evidence-based interventions in LMICs (Collins *et al*. 2011; Patel, 2012).

The argument for establishing intervention effectiveness through RCTs ensures that users are offered services which (quantitatively) reduce the target outcome, and consequently, avoid detrimental impact or waste of resources if there is no impact at all. The main strength of RCTs is their excellent internal validity due to randomization, which ensures that the only difference between the two treatment arms that are compared is their exposure to the treatment of interest. Evidence from RCTs is considered ‘gold standards’. For example, compilations such as *Blueprints for Violence Prevention* rank mental health interventions based on the number of RCTs demonstrating their efficacy/effectiveness, thus providing a useful tool for guiding large-scale implementation decisions.

In this commentary, we briefly argue that although RCTs have contributed immensely to development of mental health services in the last 50 years, positivistic experimental paradigms have several limitations. Other methods with greater external validity (or greater potential for generalizability) should also be considered for determining impact of interventions. More importantly, we discuss whether RCTs are efficient tools given the fast pace at which the society changes. In this discussion, we are not intending to undermine the value of RCTs, but rather to point out some of its limitations and recognize the benefits of other alternative methodologies for establishing intervention impact. In light of this global mental health movement, the present discussion is both relevant to efforts in high-income countries and LMICs.

## What are some of the limitations of RCTs in mental health research?

Many scholars, mainly from the medical field, have previously recognized the limitations of RCTs (e.g. Hannan, 2008; Booth & Tannock, 2014). Some limitations of a purely positivistic academic perspective are that it:

(1) dismisses techniques and practices with greater external validity, such as observational studies and ethnographic work;
(2) reduces changes (and impact) to those measured by a set of questionnaires, thus failing to acknowledge that any given behavior is the result of a complex set of interactions with others around as well as with very specific environmental circumstances;
(3) assumes that experimental knowledge gathered from a very specific context is applicable and generalizable to different settings; and thus
(4) usually fails to acknowledge the value of context and culture.

Evidence from clinical reflections, observations, ethnographic work, case studies and qualitative data are rarely taken into account when assessing the impact of an intervention, even though they have greater external validity than RCTs and are useful for acknowledging complexity of outcomes. The need to simplify outcomes in RCTs not only affects evaluation research but also influences intervention design. Interventions are becoming simpler, briefer and mainly focalized on one or two risk factors (i.e. those that can be measured and quantified). In addition, regardless of the low external validity of RCTs, interventions tend to be disseminated widely across communities, countries and cultures. Even though wide dissemination might be considered cost-effective, local culture and context-specific factors tend to be ignored when making these decisions. More importantly, once RCTs have been conducted and interventions proven impactful, its content and shape ‘freezes’ in time: any major change to the intervention might revoke its *evidence-based* status, and new funding and effort is required to establish updated experimental impact. *Freezing* interventions in space and time is imposed through strict certifications, adherence protocols and fidelity checks. Therefore, granting *evidence-based* status based on results from RCTs assumes that different communities and contexts are identical (and what works somewhere would work elsewhere) and fails to acknowledge that societies evolve rapidly in time.

## Are RCTs an efficient tool in a rapidly changing world?

Our work has mainly centered on the prevention of drug and alcohol use during childhood and adolescence in Latin America, and thus, the following examples will be specific to this issue. One example of the rapid evolution of society is the changes in marijuana use legislation around the world (e.g. legalization of marijuana in some states of the USA and in Uruguay, for example). These rapid societal changes are modifying perceptions of risk and attitudes about this drug (Schuermeyer *et al*. 2014). Current policies are also reducing the credibility of preventive interventions (Haggerty *et al*. 2015) and thus organically changing content of many interventions and the perceived need for their implementation.

**Fig. 1.**
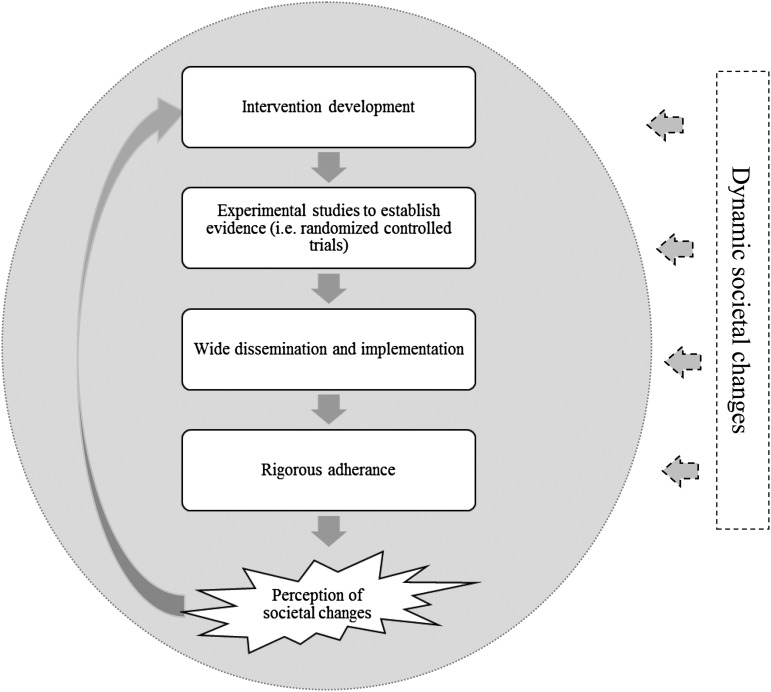
Diagrammatic conceptualization of the *evidence-based* cycle. Note 1. The gray sphere delineates what can be measured and tested empirically. Note 2. The process of establishing evidence is rigid and iterative. Dynamic societal changes (e.g. needs, structures and attitudes) are difficult to measure from a fully positivistic stance. These changes and the methods that could be used to understand them (e.g. experiential, qualitative and ethnographic data) are left out in the cycle of establishing evidence for interventions. Once societal changes are perceived by developers and policy makers, interventions are adapted and the process for establishing evidence starts once again.

One well-known intervention is the Life Skills Training Program (LST), which has been commonly implemented in both high- and low-income countries (Botvin & Griffin, 2004, 2015). This intervention is designed to improve social skills such as communication and emotional regulation in adolescents in order to prevent mental health difficulties later in life (e.g. substance use). These skills seem universal and applicable to most countries and cultures. However, there is no empirical evidence suggesting that the skills are indeed universal, and given that RCTs of the intervention have been mainly conducted in the USA, it is impossible to establish generalizability of findings. Most importantly, LST was developed 30 years ago, when adolescents had a very different pace of living, different concerns and environmental influences. There are currently no studies evaluating if the intervention is still relevant to the needs of adolescents today. In addition, recent RCTs of LST report small or no effects at all (Vicary *et al*. 2004, 2006; Gorman, 2011; Luna-Adame *et al*. 2013). A potential explanation is that the evidence of efficacy from previous RCTs might have been fixated to a very specific context and point in time. If developers incorporate changes in the original intervention to ‘update’ its content and techniques, then a new wave of expensive RCTs will be needed to grant *evidence-based* status. By the time this experimental information is available, society would have probably evolved and new changes to the content of the intervention will be needed (as well as new evidence of efficacy). In Fig. 1, we provided a diagrammatic conceptualization of this vicious cycle of gathering experimental evidence that is not necessarily timed around societal changes.

Our work with the Strengthening Families Program 10–14 (SFP 10–14; Molgaard *et al*. 1997) in Panama provides an example of how qualitative information can overcome some of these limitations. The SFP 10–14 is an evidence-based intervention (often listed in compilations as one with ‘promising evidence’) with several trials documenting its effectiveness, most of which have been conducted in the USA. In between 2010 and 2012, the United Nations Office on Drugs and Crime invested in training facilitators to deliver the intervention in Panama in order to prevent adolescent substance use. Instead of conducting an RCT, we opted for evaluating the appropriateness of its content in this context, its potential for implementation and parents’ experiences after taking part in the intervention using qualitative methods (Mejia *et al*. 2014, 2015). Although our methodology might not be sufficient for establishing efficacy/effectiveness based on current guidelines, it allowed an in-depth, participant-driven, comprehensive understanding of the impact of the intervention in these communities. We were also able to gather practical information that could inform larger-scale implementation. This would not have been possible by solely conducting an RCT.

## Conclusion

In this brief commentary, we discussed a never-ending chain of rigorous (and rigid) knowledge in mental health that tends to be applicable to a specific time and space. Although efforts to promote experimental evaluations are respectable and have allowed significant progress in the field, RCTs have often decreased flexibility in the content of interventions and have failed to recognize the impact of societal changes. We propose acknowledging alternative methodologies, such as observational and naturalistic studies, case studies and qualitative data. Two case examples to support this argument were provided.
